# Phytosomes as a Plausible Nano-Delivery System for Enhanced Oral Bioavailability and Improved Hepatoprotective Activity of Silymarin

**DOI:** 10.3390/ph15070790

**Published:** 2022-06-24

**Authors:** Ravi Gundadka Shriram, Afrasim Moin, Hadil Faris Alotaibi, El-Sayed Khafagy, Ahmed Al Saqr, Amr Selim Abu Lila, Rompicherla Narayana Charyulu

**Affiliations:** 1 Department of Pharmaceutics, NGSM Institute of Pharmaceutical Sciences, Nitte (Deemed to be University), Mangalore 575018, Karnataka, India; ravigs1991@gmail.com; 2 Department of Pharmaceutics, College of Pharmacy, University of Hail, Hail 81442, Saudi Arabia; a.moinuddin@uoh.edu.sa; 3 Department of Pharmaceutical Sciences, College of Pharmacy, Princess Nourah bint Abdul Rahman University, Riyadh 11671, Saudi Arabia; hfalotaibi@pnu.edu.sa; 4 Department of Pharmaceutics, College of Pharmacy, Prince Sattam bin Abdulaziz University, Al-Kharj 11942, Saudi Arabia; e.khafagy@psau.edu.sa (E.-S.K.); a.alsaqr@psau.edu.sa (A.A.S.); 5 Department of Pharmaceutics and Industrial Pharmacy, Faculty of Pharmacy, Suez Canal University, Ismailia 41522, Egypt; 6 Department of Pharmaceutics and Industrial Pharmacy, Faculty of Pharmacy, Zagazig University, Zagazig 44519, Egypt

**Keywords:** anti-oxidant activity, hepatoprotective effect, phospholipid, phytosomes, Silymarin

## Abstract

Silymarin, a phyto-constituent derived from the plant *Silybum marianum*, has been widely acknowledged for its hepatoprotective activities. Nevertheless, its clinical utility is adversely hampered by its poor water-solubility and its limited oral bioavailability. The aim of this study was to investigate the efficacy of phospholipid-based phytosomes for enhancing the oral bioavailability of silymarin. The phytosomes were prepared using the solvent evaporation technique and were optimized using a full factorial design. The optimized silymarin phytosomal formulation was then characterized for particle size, surface morphology, aqueous solubility, and in vitro drug release. Furthermore, in vivo antioxidant activity, hepatoprotective activity and oral bioavailability of the optimized formula were investigated in a rat model. The prepared silymarin phytosomes were discrete particles with a porous, nearly smooth surface and were 218.4 ± 2.54 nm in diameter. In addition, the optimized silymarin phytosomal formulation showed a significant improvement in aqueous solubility (~360 µg/mL) compared to pure silymarin and manifested a higher rate and extent of silymarin release from the optimized formula in dissolution studies. The in vivo assessment studies revealed that the optimized silymarin phytosomal formulation efficiently exerted a hepatoprotective effect in a CCl_4_-induced hepatotoxicity rat model via restoring the normal levels of antioxidant enzymes and ameliorating cellular abnormalities caused by CCl_4_-intoxication. Most notably, as compared to pure silymarin, the optimized silymarin phytosomal formulation significantly improved silymarin oral bioavailability, as indicated by a 6-fold increase in the systemic bioavailability. Collectively, phytosomes might represent a plausible phospholipid-based nanocarrier for improving the oral bioavailability of phyto-constituents with poor aqueous solubility.

## 1. Introduction

Traditionally, herbal medications, often known as phyto-pharmaceuticals, had been widely used in many countries for the management and treatment of many health disorders [1]. Globally, herbal medicine has gained cumulative popularity in modern medical practice because of their availability along with their diverse therapeutic applications [2]. Nevertheless, despite the fact that plant extracts and phyto-constituents may exert excellent in vitro bioactivity, they usually show poor in vivo effects due to their large molecular sizes and/or low lipid solubility, making them less absorbable and having poor bioavailability [3,4].

Silymarin is a natural polyphenolic flavonoid compound extracted from milk thistle (*Silybum marianum*) seeds [5]. Silymarin has been proved to exert supreme therapeutic activity in the treatment of a variety of liver disorders such as chronic liver disease, cirrhosis, and hepatocellular carcinoma [6]. In addition, a mounting body of literature has emphasized the therapeutic potential of silymarin as an anti-inflammatory, antioxidant, hypoglycemic, anticancer, and antiviral agent [7,8]. Furthermore, recent encouraging results have underscored the neuroprotective effect of silymarin in the management of neurodegenerative diseases such as traumatic brain injury and Alzheimer’s disease [9,10]. Nevertheless, despite clinical trials demonstrating that silymarin is safe at large dosages, up to 1500 mg/day in humans, it has limitations such as limited water-solubility, poor bioavailability, and poor intestinal absorption [11], which collectively could constrain its widespread utilization in many clinical settings.

The application of nanotechnology appears to be a potential way to amplify the therapeutic activity of the active herbal extract via improving its bioavailability and promoting a prolonged drug release at the site of absorption. Many strategies have been adopted to enhance the aqueous solubility and the systemic bioavailability of active phyto-constituents following oral administration, including nanoemulsion, solid lipid nanoparticles, polymeric nanoparticles, liposomes, inclusion complexation, etc. [11,12,13,14]. Among these potential strategies, phytosomes, also known as phyto-phospholipid complexes, have emerged as an encouraging strategy to improve the bioavailability of active phyto-constituents. Phytosomes are vesicular drug delivery systems that could enhance the absorption and bioavailability of poorly water-soluble drugs [15]. They are prepared by complexing the naturally active phyto-constituent with phospholipids [3]. Unlike other lipid-based vesicular systems, the bioactive phyto-constituents represents an integral part of vesicular membrane by being anchored to the polar head of the phospholipid via a chemical (hydrogen) bond, rather than been entrapped within the aqueous core or phospholipid bilayers of vesicular membrane. Importantly, phytosomes offer several advantages, including increased drug encapsulation, improved stability (chemical bonds are formed between the polar head of the amphiphile molecule and the phytoconstituent), and improved bioavailability [16]. In addition, a faster absorption rate results in a smaller dose of active components required to exert the intended pharmacological effect. Telange et al. [17] reported that loading the polyphenolic flavonoid apigenin onto a phytosomal formulation significantly enhanced its aqueous solubility, and oral bioavailability and showed a superior hepatoprotective effect, compared to pure apigenin. Similarly, Rathee et al. [18] evaluated the antidiabetic potential of polyherbal extracts loaded onto phosphatidylcholine-based phytosomes. The authors demonstrated that polyherbal extract-loaded phytosomes efficiently induced remarkable antidiabetic activity in streptozotocin-nicotinamide-induced rat models, which was comparable to that of a standard hypoglycemic drug metformin.

The aim of the present work was to enhance the absorption and oral bioavailability of silymarin via its formulation within a phytosomal nanocarrier system. A two-factor, three-level full factorial design was adopted to formulate and optimize silymarin-loaded phytosomes. The optimized formulation showed porous, nearly smooth surface particles within the nano-size range. In addition, the optimized formula showed a remarkable improvement in aqueous solubility of loaded silymarin, compared to pure silymarin. Most importantly, the optimized silymarin phytosomal formulation efficiently improved silymarin oral bioavailability and exerted a superior hepatoprotective effect in a CCl_4_-induced hepatotoxicity rat model, compared to plain silymarin.

## 2. Results and Discussion

### 2.1. Preparation of Silymarin Phytosomal Complex

The stable silymarin phytosomal complex with the phospholipid was formulated using the solvent evaporation method. A preliminary examination of the investigated process parameters indicated that the drug-to-phospholipid and reaction temperature had a significant impact on the particle size and drug content of the produced phytosomes. Accordingly, a three-level, two-factor full factorial design was adopted for the formulation and optimization of the silymarin phytosomal complex. In the current study, a total of 9 runs (Table 1) were formulated by altering two independent formulation variables; drug-to-phospholipid (X_1_) and reaction temperature (X_2_) and their impact on two dependent formulation parameters; particle size (Y_1_) and drug content (Y_2_) was assessed. The estimated values from the experimental trials revealed that the particle size fluctuated from 220.2 ± 1.27 to 494.2 ± 6.64 nm, while the drug content ranged from 67.3 ± 2.64 to 92.4 ± 3.51%. The fitted polynomial equations relating the responses (particle size and drug content) to the altered formulation variables are summarized in the following equations:

Y_1_ = 222.14 + 29.47 X_1_ − 49.43 X_2_ + 2.02 X_1_X_2_ + 58.43 X_1_^2^ + 133.83 X_2_^2^

Y_2_ = 91.38 − 0.133 X_1_ + 3.62 X_2_ − 2.25 X_1_X_2_ − 3.57 X_1_^2^ − 11.32 X_2_^2^

The obtained polynomial equations can be utilized to extract conclusions based on the magnitude of coefficient and the mathematical sign it carries. A negative sign signifies an antagonistic effect, whilst a positive sign signifies a synergistic effect of the factor on the selected response.

**Figure 1:**
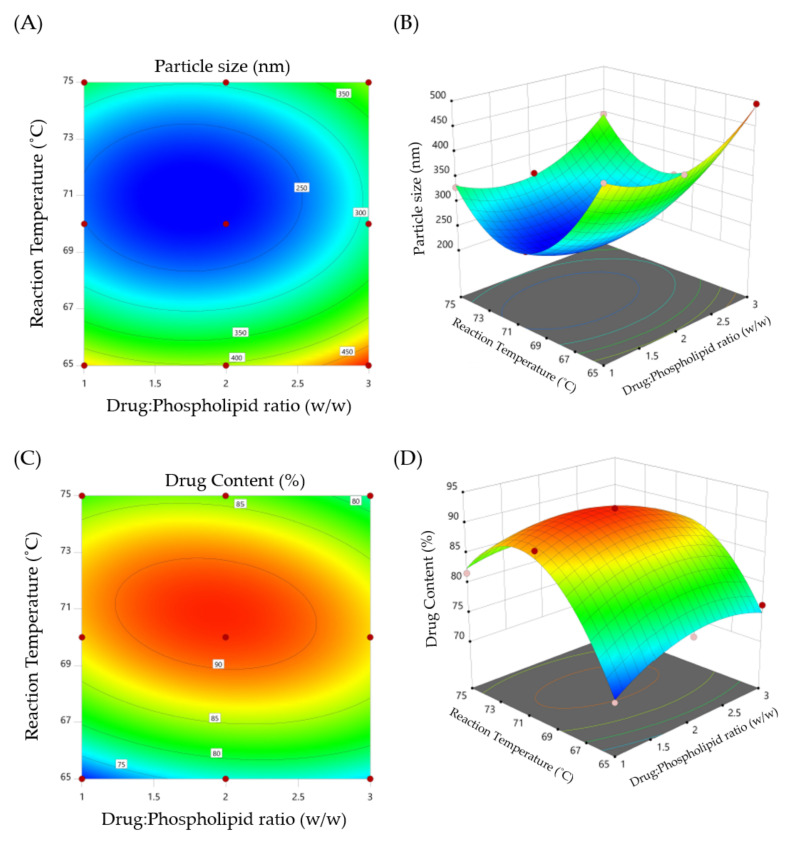
(**A**) Contour plot of particle size (Y_1_); (**B**) 3D surface plot for Y_1_; (**C**) contour plot of drug content (Y_2_); and (**D**) 3D surface plot for Y_2_.

Response surface plots and contour plots were also used to determine the significance and amplitude of the tested dependent factors on the independent responses (Figure 1). The response surface and contour plots revealed that the studied parameters; drug-to-phospholipid (X_1_) and reaction temperature (X_2_), had a significant impact on both formulation responses; particle size (Y_1_) and drug content (Y_2_). It was evident that the particle size decreased as the drug:phospholipid ratio increased from 1:1 to 1:2. A further increase in the ratio to 1:3 was found to increase the particle size. Similarly, the drug content (%) was found to be increased upon increasing drug:phospholipid ratio from 1:1 to 1:2. A further increase in drug:phospholipid ratio to 1:3 significantly decreased the drug content. In addition, the reaction temperature exerted significant effects on both particle size and drug content. Phytosomes prepared at a reaction temperature of 70 °C showed the smallest particle size, and the highest drug content, compared to phytosomes prepared at a higher reaction temperature (75 °C). Similar findings were reported by Telange et al. who demonstrated that both drug:phospholipid ratio and reaction temperature could significantly affect the entrapment efficiency of the polyphenolic flavonoid, apigenin, within soybean phosphatidylcholine-based phytosomes [17].

### 2.2. Optimization of Silymarin Phytosomal Complex

A desirability approach was adopted to obtain an optimized phytosomal formulation with desired responses, such as minimum particle size and maximum drug content. Out of the generated solutions, the optimized formula was selected based on the desirability values (near to 1). The optimized phytosomal formula selected under the aforementioned constrains was obtained at a drug:phospholipid ratio of 1:1.93 and a reaction temperature of 70.8 °C. The particle size and drug content (%) of the formulated optimized silymarin phytosomal complex were 218.4 ± 2.54 nm and 90.21 ± 4.03%, respectively, which were close to predicted values (215.8 nm and 91.68%) of the phytosomal complex obtained at a desirability value of 0.984.

### 2.3. Evaluation of Silymarin Phytosomal Complex

#### 2.3.1. Average Particle Size, Polydispersity Index and Zeta Potential

Particle size, PDI, and zeta potential are considered the main parameters that dictate the effective distribution and physical stability of the lipidic nanocarrier systems in a liquid medium [19]. In this study, the optimized silymarin phytosomes had a particle size of 218.4 ± 2.54 nm with PDI of 0.256 ± 0.02, indicating a narrow range of particle size distribution (Figure S1).

Zeta potential is a key determinant of stability of colloidal dispersions. It has been reported that a zeta potential value greater than ±30 mV is desired for an electrostatically stable formulation [20]. Zeta potential relies on the type and composition of the phospholipid used in the formulation. The optimized silymarin phytosomes had a zeta potential value of −30.8 mV (Figure S2), indicating good physical stability of the formulated phytosomes. Such relatively high zeta potential was attributed to the presence of a negatively charged phosphate group in the polar head of the phospholipid [21].

#### 2.3.2. Surface Morphology

**Figure 2:**
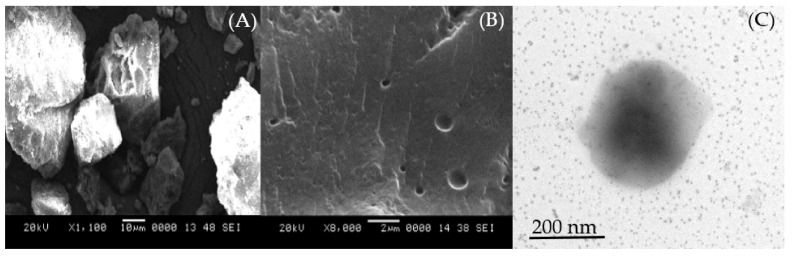
SEM image of (**A**) silymarin and (**B**) optimized silymarin phytosomes. (**C**) TEM image of optimized silymarin phytosomes.

The surface morphology of the optimized silymarin phytosomal formulation was studied using SEM and TEM analysis. The SEM image of optimized silymarin phytosomes disclosed discrete particles with a porous and nearly smooth surface (Figure 2A), compared to the crystalline surface of pure silymarin (Figure 2B). This might account for the improved solubility of phytosomal vesicles compared to the pure drug. Further, when these optimized silymarin phytosomes were dispersed in distilled water, vesicular nanostructures were formed without any aggregation or decomposition, as evidenced by TEM images (Figure 2C), suggesting the formation of well-formed discrete vesicles.

### 2.4. Structural Characterization Silymarin Phytosomal Complex

#### 2.4.1. Fourier Transform Infrared Spectroscopy (FTIR)

**Figure 3:**
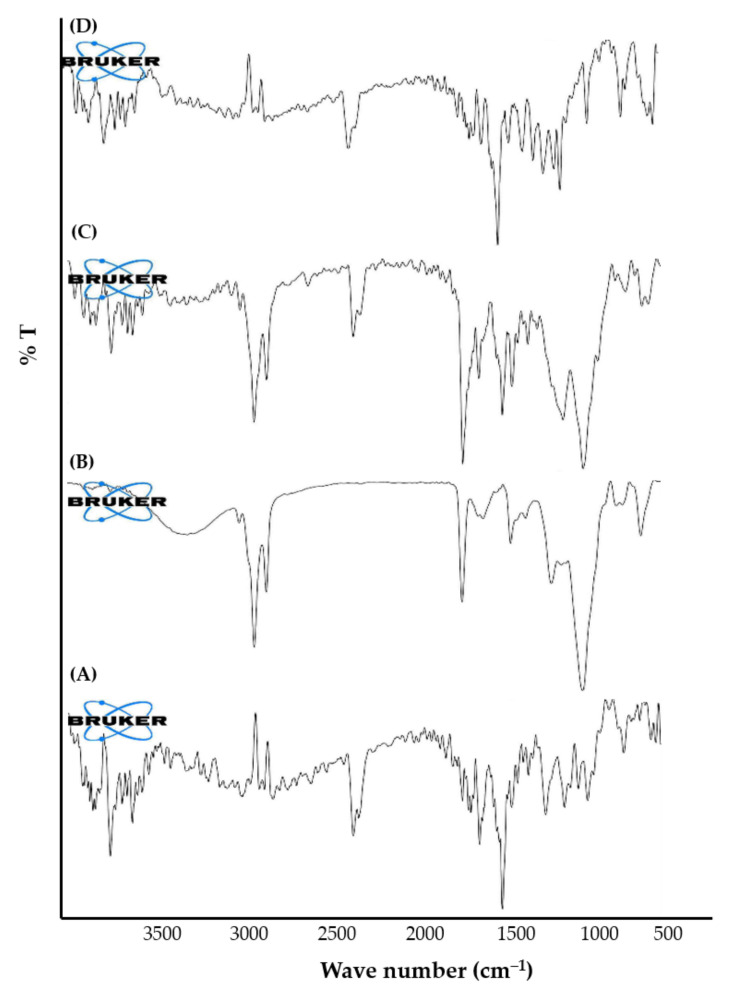
FTIR spectra of (**A**) silymarin; (**B**) SPC; (**C**) physical mixture of silymarin + SPC; and (**D**) optimized silymarin phytosomal formulation.

FTIR spectroscopy is a valuable tool for identifying the interaction between different components in the same formulation. Accordingly, the formation of the silymarin-phospholipid phytosomal complex was confirmed by the FTIR spectroscopy via matching the spectrum of the complex with the spectra of individual components used for the preparation of phytosomes. The FTIR spectra of silymarin, SPC, physical mixture of silymarin + SPC, and optimized silymarin phytosomal formulation are depicted in Figure 3. The FTIR spectrum of silymarin showed characteristic absorption peaks at 3647 cm^−1^ (O—H), 2876 cm^−1^ (C—H), 1643 cm^−1^ (C=O), and 1513 cm^−1^ (aromatic C=C). The FTIR spectrum of SPC unveiled characteristic absorption peaks at 2922 cm^−1^ and 2853 cm^−1^ (C—H, fatty acid chain), 1737 cm^−1^ (C=O, fatty acid ester), 1227 cm^−1^ (P=O), and 1048 cm^−1^ (P—O—C). The spectrum of physical mixture of silymarin + SPC retained almost all the characteristic peaks of individual components. Of interest, remarkable changes were observed in the spectrum of the optimized silymarin phytosomal formulation. Broadening of the stretching absorption band of phenolic (O—H) of silymarin, along with the disappearance of characteristic absorption peaks at 1227 cm^−1^ and 1048 cm^−1^ for SPC in the phytosomal formulation suggests the occurrence of weak intermolecular interactions (H-bonding formation) between silymarin and SPC during the formation of the phytosomal complex. These results are in alignment with that of Hooresfand et al. [22], who demonstrated the formation of a weak bond between rutin and phospholipids during the formation of drug-phospholipid phytosomal complexes.

#### 2.4.2. Differential Scanning Calorimetry (DSC)

**Figure 4:**
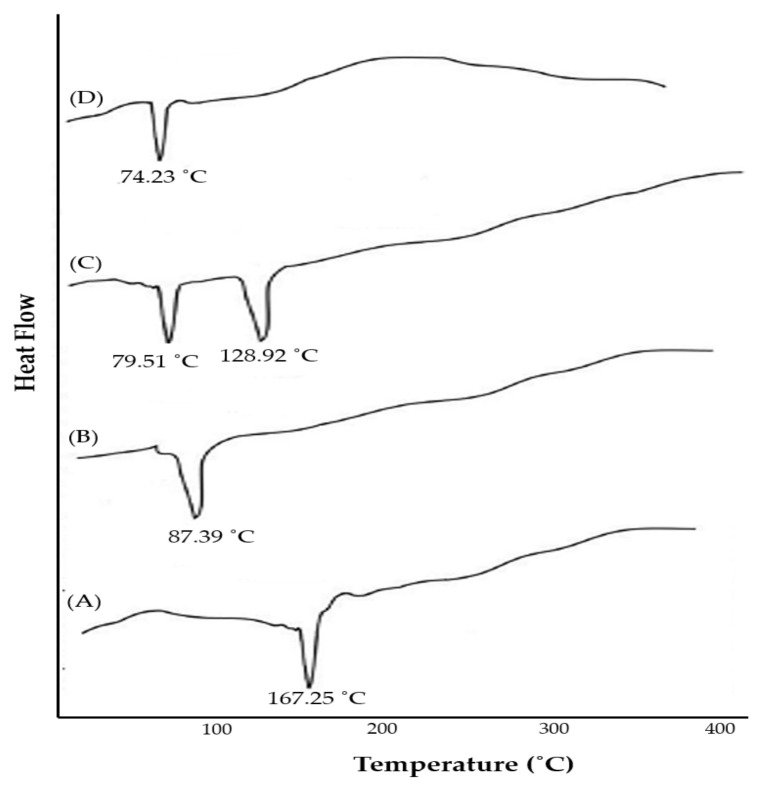
DSC thermograms of (**A**) silymarin; (**B**) SPC; (**C**) physical mixture of silymarin + SPC; and (**D**) optimized silymarin phytosomal formulation.

Thermal analysis is a widely used approach to characterize the solid-state matter in the complex form. In differential scanning calorimetry (DSC), determination of changes in solid-state properties with respect to temperature change could provide useful information regarding the stability, degradation, and melting of tested materials [17]. Most importantly, DSC analysis could also afford information regarding drug-excipient interactions. DSC thermograms of silymarin, SPC, physical mixture of silymarin + SPC, and optimized silymarin phytosomal formulation are depicted in Figure 4. The thermogram of silymarin (Figure 4A) exhibited a sharp endothermal melting peak at 167.25 °C, indicating the crystalline nature of pure silymarin. The thermogram of SPC showed a sharp endothermal peak at 87.39 °C (Figure 4B) corresponding to the gel-to-liquid crystal state transition [23]. The thermogram of the physical mixture of silymarin + SPC (Figure 4C) showed a remarkable shift in the endothermic peaks of both silymarin and SPC towards lower temperatures (128.92 °C, and 79.51 °C, respectively). Of interest, the thermogram of the optimized silymarin phytosomal complex revealed the disappearance of the sharp endothermal peaks of both silymarin and SPC, and the appearance of a new peak broad endothermal peak at 74.23 °C. These results suggest that a stable silymarin-phospholipid phytosomal complex was formed via weak intermolecular interactions, van der Waals interactions, and/or hydrogen bonding, between silymarin and SPC. These interactions may allow the fatty acid chains of phospholipid to freely spin and enwrap silymarin molecules at a molecular level [24].

#### 2.4.3. X-ray Powder Diffractometry (XRD)

**Figure 5:**
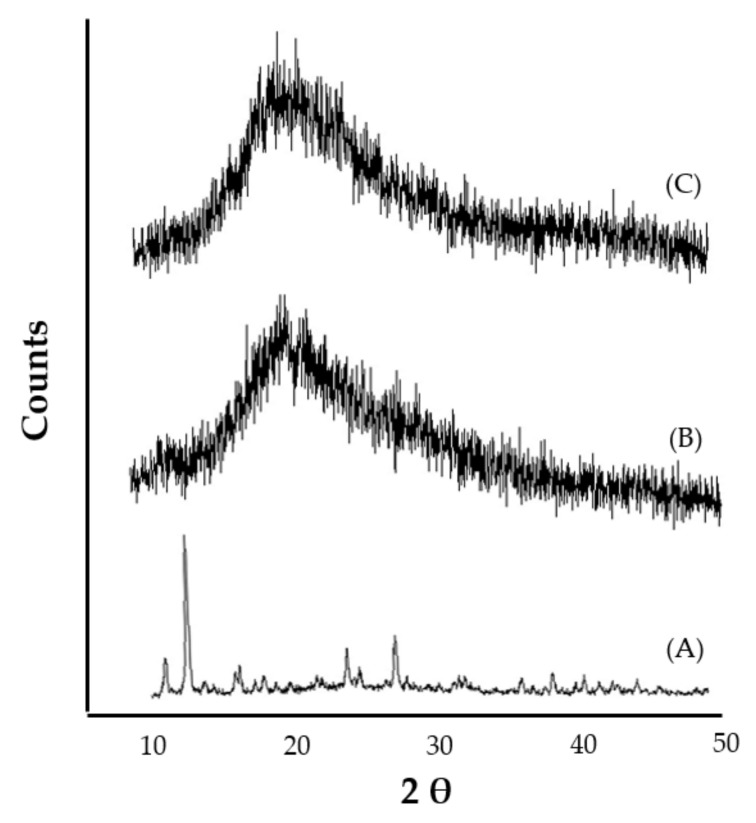
X-ray diffractograms of (**A**) silymarin; (**B**) SPC; and (**C**) optimized silymarin phytosomal formulation.

The molecular crystallinity of the optimized silymarin phytosomes was determined using the X-ray powder diffractometry technique. The X-ray diffractograms of silymarin, SPC, and the optimized silymarin phytosomal formulation are depicted in Figure 5. The X-ray diffractogram of silymarin exhibited intense and sharp peaks at 2θ = 13.16°, 14.45°, 22.56°, and 26.74°, indicating the crystalline nature of silymarin (Figure 5A). On the other hand, the X-ray diffractogram of SPC manifests a single, relatively broad diffraction peak at 2θ = 20.05°, indicating the amorphous nature of SPC (Figure 5B). Of interest, the X-ray diffractogram of the optimized silymarin phytosomal formulation showed a single broad peak at 2θ = 21.13° (Figure 5C), which was similar to that of SPC, suggesting that silymarin in the optimized phytosomal formula is molecularly dispersed in a phospholipid matrix in the amorphous form. Similar results were reported by Cai et al., who demonstrated the change in the crystallinity of the cholinesterase inhibitor, huperzine A, from the crystalline state to the amorphous state upon complexing with phospholipids [25].

### 2.5. Solubility Study

Solubility and partition coefficients are two crucial factors that dictate the in vivo fate of orally administered drugs. Generally, orally administered drugs cannot be overly lipophilic as this will lead to poor absorption. Silymarin is a hydrophobic compound that shows very poor bioavailability due to its poor water solubility [26]. The results of aqueous solubility of pure silymarin and the silymarin-phospholipid phytosomal complex are summarized in Table 2. As depicted in Table 2, silymarin shows poor aqueous solubility in distilled water (45.7 µg/mL). On the other hand, a significant increase was observed in the aqueous solubility of the silymarin phytosomal complex (358.8 µg/mL) in comparison with pure silymarin (*p* < 0.001). This increased solubility of the silymarin phytosomal formulation might be attributed, on the one hand, to the change in drug crystallinity to the amorphous state upon complexing with phospholipid as confirmed by X-ray diffraction analysis, and on the other hand, to the amphiphilic nature of phytosomal formulation.

Generally, drugs with balanced water solubility and lipid solubility could efficiently penetrate the cell membrane lipid bilayer and thereby exert their pharmacological actions. Besides its enhancing effect on the aqueous solubility of silymarin, the phytosomal formulation was found to enhance the lipid solubility of silymarin as well. The silymarin phytosomal formulation showed a ~4.5-fold increase in lipid (n-octanol) solubility, compared to the pure drug (Table 2). The enhanced lipid solubility of silymarin formulated within phytosomes might be ascribed to the engagement of polar heads of the drug and phospholipid in the complex (H-bond) formation, whilst the two long fatty chains of phospholipid molecules did not engage in the complex process and were freely rotatable, forming a lipophilic surface that bestowed the silymarin phytosomal formulation with lipid soluble characteristics [27]. Collectively, these results suggest the efficacy of silymarin-phospholipid phytosomal complexes in, not only enhancing the aqueous solubility of the lipophilic drug, silymarin, but in lipid solubility as well, promoting higher drug permeation through biological membranes with subsequent improvements in the oral bioavailability of the drug.

### 2.6. Dissolution Study

**Figure 6:**
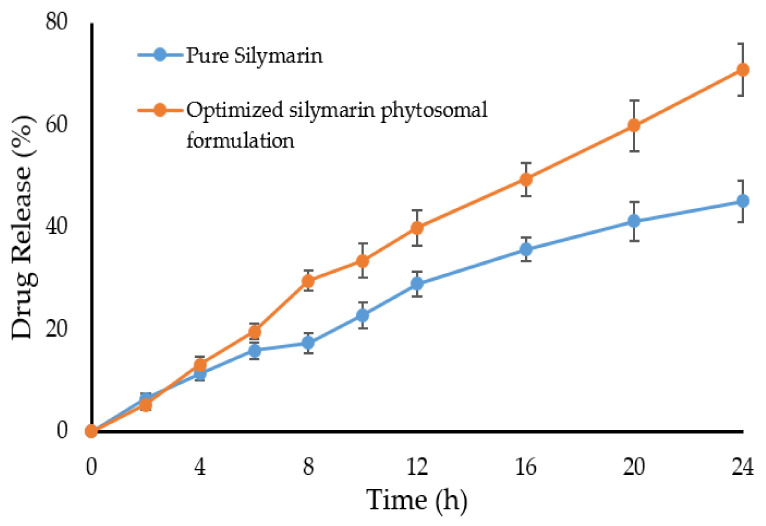
In vitro drug release profile of pure silymarin and optimized silymarin phytosomal formulation in phosphate buffer pH 7.4. Values are mean ± SD (*n* = 3).

Figure 6 shows the dissolution profiles of pure silymarin and the optimized silymarin phytosomal formulation in a phosphate buffer pH 7.4. It was observed that the drug release pattern of pure silymarin and the optimized silymarin phytosomal formulation was similar up to 8 h. After 8 h, a plateau state was observed in the case of pure silymarin showing a maximum of 45% drug released at the end of 24 h. Unlike pure silymarin, the optimized silymarin phytosomal formulation exhibited sustained drug release; reaching 70.8% at the end of 24 h. The increased drug release from the optimized phytosomal formulation might be ascribed to the physicochemical changes that occurred upon complexing the drug with phospholipid, which increased the solubility of the complex compared to the pure silymarin, as evidenced by in vitro solubility studies.

To gain an insight into the release kinetics of silymarin from the silymarin phytosomal formulation, the release data were fitted into different kinetic models. In vitro release data revealed that drug release from the phytosomal formulation followed the Higuchi model; indicating that drug release is diffusion controlled.

### 2.7. In Vivo Hepatoprotective Effect of Silymarin-Phospholipid Phytosomal Complex

The liver is the major organ of metabolism and excretion that is involved in the detoxification process. Liver damage is one of the major health problems that is caused by various hepatotoxins. Carbon tetrachloride (CCl_4_) is a hepatotoxin that has been commonly utilized in animal studies. In vivo, CCl_4_ is converted by hepatic CYP450 enzymes producing reactive oxidant species that harm important organs such as the liver, kidney, heart, and brain [28], and reduce the activity of serum antioxidant enzymes via the generation of robust amounts of free radicals, which ultimately leads to the lipid peroxidation of cellular membranes. In the present study, hepatotoxicity was induced in the rats by using CCl_4_, and the hepatoprotective effect of either pure silymarin or silymarin-phospholipid phytosomal complexes, in terms of normalizing serum levels of hepatic markers, was investigated. As summarized in Table 3, the serum levels of hepatic marker enzymes such as SGPT, SGOT, SALP, and total bilirubin were significantly elevated (*p* < 0.01), compared to non-CCL_4_ intoxicated normal rats, confirming the hepatic damage caused by CCl_4_. On the other hand, pre-treatment with pure silymarin for 7 days remarkably protected the animals from the hepatotoxic effect of CCl_4_ as manifested by a considerable decrease in serum levels of marker enzymes, compared to CCl_4_-intoxicated rats. Of interest, pre-treatment of animals with silymarin-phospholipid phytosomal complexes for 7 consecutive days nearly restored the serum levels of tested hepatic markers to the normal levels of negative control group (Table 3). These findings imply the superior hepatoprotective effect of silymarin phytosomes compared to that of pure silymarin.

### 2.8. In Vivo Antioxidant Activity of Silymarin-Phospholipid Phytosomal Complex

Globally, silymarin is considered one of the most commonly used natural compounds for the treatment of hepatic diseases owing to its antioxidant, antifibrotic, and anti-inflammatory activities [29]. Accordingly, in order to address the antioxidant potential effect of silymarin-phospholipid phytosomal complex, the levels of antioxidant enzymes, namely, glutathione reductase (GRD), reduced glutathione (GSH), glutathione S transferase (GST), glutathione peroxidase (GPx), catalase (CAT), and superoxide dismutase (SOD) were assayed in liver homogenates of CCl_4_-treated and naïve rats. The effect of either pure silymarin or silymarin-phospholipid phytosomal complexes antioxidant biochemical paradigms is depicted in Table 4. It was evident that CCl_4_ intoxication ensued a significant reduction (*p* < 0.01) in the levels all the tested antioxidant enzymes in liver homogenates compared to naïve control rats. In contrast, pre-treatment with pure silymarin for 7 consecutive days substantially (*p* < 0.05) reduced the CCl_4_-induced drop in GSH, GPx, and CAT levels. Most importantly, pre-treatment with optimized silymarin phytosomes efficiently protected the animals from the CCl_4_-induced drop in all tested antioxidant enzymes. The levels of all tested antioxidant enzymes (GSH, GPx, GST, GRD, SOD, and CAT) in liver homogenates were comparable to that of the naïve negative control group. These results suggest that optimized silymarin phytosomes could efficiently exert a hepatoprotective effect against CCl_4_-induced intoxication via restoring the normal levels of antioxidant enzymes.

**Figure 7:**
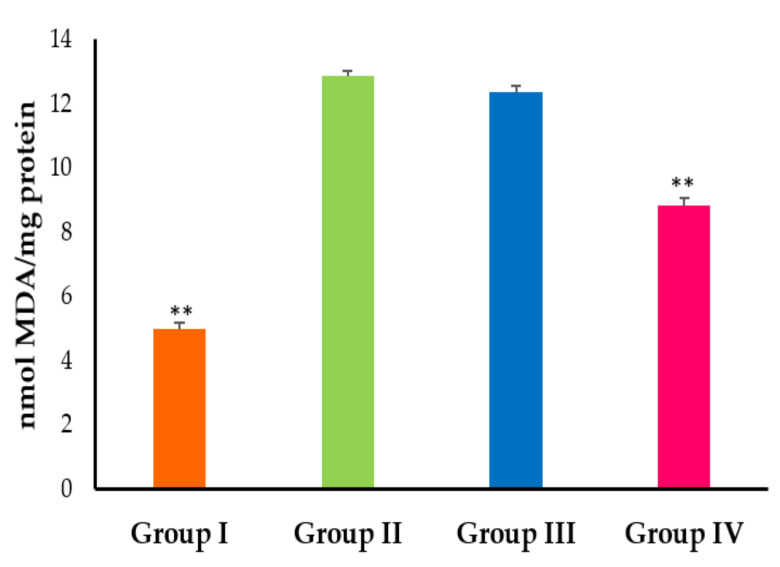
Effect of pure silymarin and optimized silymarin phytosomal formulation on lipid peroxidase (nmoles of MDA released/g tissue). Group I represents a negative control (treated with Tween 20 (1% *v*/*v*); Group II represents CCl_4_-intoxicated rats (treated with CCl_4_ + olive oil (1:1.5 mL/kg)); Group III represents CCl_4_-intoxicated rats treated with plain silymarin; and Group 4 represents CCl_4_-intoxicated rats treated with optimized silymarin phytosomal formulation. Data are mean ± SD (*n* = 6). ** *p* < 0.01 vs. CCl_4_-intoxicated rats.

To gain further insight into the antioxidant potential of silymarin-phospholipid phytosomal complexes, quantitative evaluation of malondialdehyde (MDA), a major lipid peroxidation product, was determined via thiobarbituric acid reactive substances (TBARS) assays [30]. As shown in Figure 7, CCl_4_ intoxication triggered potent lipid peroxidation (LPO), as manifested by significantly (*p* < 0.01) elevated levels of MDA in CCL_4_-intoxicated rats compared to naïve normal rats. In addition, pre-treatment with pure silymarin failed to protect the rats from CCL_4_-triggred lipid peroxidation, as evidenced by comparable levels of MDA in both silymarin-pretreated CCL_4_-intoxicated rats and positive control (CCL_4_-intoxicated) rats. On the other hand, pre-treatment with silymarin-phospholipid phytosomal complexes significantly (*p* < 0.01) abrogated the CCl_4_-induced increase of MDA levels. Cytochrome P 450-dependent monooxygenases is known to process the accumulated CCl_4_ to trichloromethyl (CCl_3_) radicals in the hepatic parenchymal cells [31]. Besides its role in the alkylation of cellular proteins, CCl_3_ causes the polyunsaturated fatty acids to produce lipid peroxides, which could induce hepatotoxicity and alter hepatic marker enzyme levels [32,33]. In this study, the optimized silymarin phytosomal formulation showed the potential to ameliorate all cellular changes induced by CCl_4_-intoxication. The optimized silymarin phytosomal efficiently restored all CCl_4_-elevated rat liver function marker enzymes, resisted the CCl_4_-induced reduction in antioxidant enzymes and significantly abrogated the CCl_4_-induced increase in MDA levels. Collectively, these results underscore the reactive oxygen species (ROS) scavenging ability of silymarin phytosomal formulation, which efficiently helps in overcoming the oxidative damage/stress elicited by CCl_4_-intoxication in rat models.

### 2.9. Histopathological Studies

**Figure 8:**
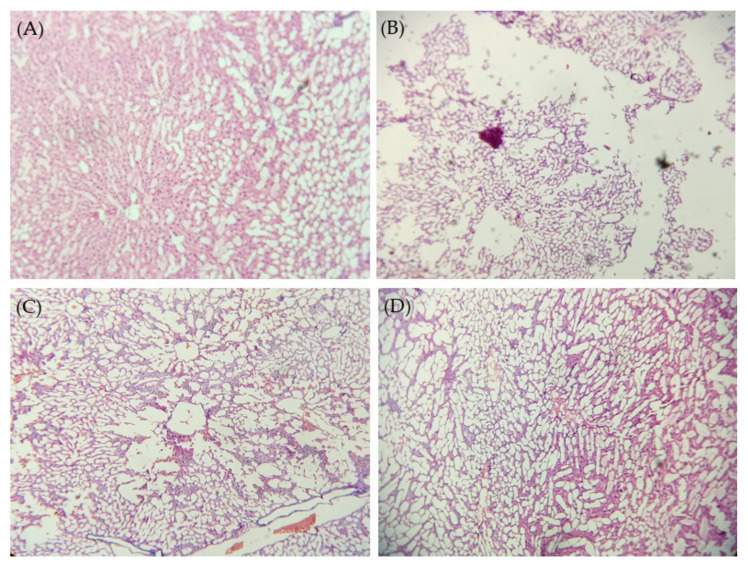
Histological micrographs of liver tissue of (**A**) negative control (treated with Tween 20 (1% *v*/*v*); (**B**) CCl_4_-intoxicated rats (treated with CCl_4_ + olive oil (1:1.5 mL/kg)); (**C**) CCl_4_-intoxicated rats treated with plain silymarin and (**D**) CCl_4_-intoxicated rats treated with optimized silymarin phytosomal formulation. 100× magnification.

Histological examination of rat liver tissues was adopted to assess the effect of pure silymarin or the optimized silymarin phytosomal formulation on CCl_4_-induced liver damage. As shown in Figure 8, liver tissue of non CCl_4_-intoxicated control rats showed well-preserved cellular structure with clear cytoplasm, indicating healthy functional liver cells. On the other hand, in CCl_4_-intoxicated rats, obvious degeneration of parenchymal cells and fatty tissues with severe damage in the central lobular area was observed, underscoring the hepatotoxic effect of CCl_4_. Pre-treatment with pure silymarin resulted in a moderate hepatoprotective effect as manifested by a remarkable decrease in fatty tissue degeneration and parenchymal cells damage. Of interest, the optimized silymarin phytosomal formulation efficiently protected liver tissue from the hepatotoxic effect of CCl_4_. Normal hepatic cells with a well-restored cytoplasm and central vein were observed in the liver section of silymarin phytosomes-pre-treated rats. These findings suggest that the optimized silymarin phytosomal formulation could efficiently restore the normal anatomy of hepatic cells, presumably, via the augmented antioxidant potential of silymarin.

### 2.10. Pharmacokinetics Study

**Figure 9:**
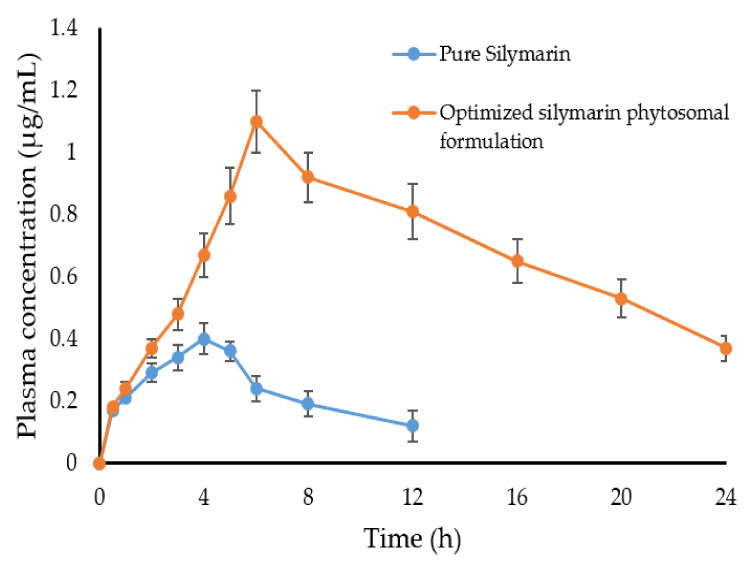
Mean plasma concentration-time profile of pure silymarin (100 mg/kg, p.o.) and optimized silymarin phytosomes (~100 mg/kg silymarin, p.o). Data are mean ± SD (*n* = 6).

To gain an insight into the underlying mechanism of the enhanced in vivo hepatoprotective effect of silymarin-phospholipid phytosomal complexes compared to pure silymarin, the in vivo pharmacokinetics of either plain silymarin (100 mg/Kg) or the optimized silymarin phytosomal formulation (100 mg/kg silymarin) were investigated in Wistar rats following oral administration. Figure 9 represents the mean plasma silymarin concentrations as a function of time. As depicted in Figure 9, plasma levels of plain silymarin were very low, presumably, due to its poor aqueous solubility, which might hinder its proper absorption. In contrast, a significant elevation in the plasma concentration of silymarin was observed in animals treated with the optimized silymarin phytosomal formulation. Such relatively higher plasma drug concentrations of silymarin from the phytosomal formulation following oral administration might be ascribed to the enhanced drug absorption from the phytosomal formulation owing to the amphiphilic nature of the formulation. Of note, phytosomes succeeded to maintain silymarin plasma concentrations at remarkably higher levels for a prolonged period of time (up to 24 h post administration).

The key pharmacokinetic parameters of silymarin are illustrated in Table 5. As depicted in Table 5, the optimized silymarin phytosomal formulation showed a significantly higher peak concentration (C_max_ = 1.1 ± 0.12 μg/mL) compared to the plain drug (C_max_ = 0.4 ± 0.10 μg/mL), indicating higher absorption of the drug from the optimized formulation. In addition, the optimized silymarin phytosomal formulation exhibited a longer half-life (t_1/2_) and mean residence time (MRT) compared to the plain drug. The MRT of plain silymarin and the optimized silymarin phytosomal formulation were 9.44 ± 1.2 h and 20.43 ± 1.8 h, respectively. Such longer residence and/or prolonged duration of action of the optimized silymarin phytosomal formulation might be ascribed to the reduced systemic clearance of the optimized silymarin phytosomal formulation (Cl = 4.48 ± 0.77 mL·h^−1^) compared to the plain drug (Cl = 26.03 ± 1.9 mL·h^−1^). Most importantly, the mean relative bioavailability of the optimized silymarin phytosomal formulation was ~6-fold that of the plain drug. Such observed improvements in silymarin relative bioavailability following oral administration of the optimized silymarin phytosomal formulation can be accredited to the presence of phospholipid, which could efficiently enhance silymarin aqueous solubility, leading to increased intestinal absorption. In addition, complexation of the drug within the amphiphilic phospholipid-based vesicular carrier was reported to shield the drug from hepatic first-pass metabolism, and thereby, enhance its systemic bioavailability [17].

## 3. Materials and Methods

### 3.1. Materials

Soybean phosphatidyl choline (Phospholipon^®^ 90 G; 98% phopsphatidylcholine content) was received as a gift sample from Lipoid GmbH (Ludwigshafen, Germany). Silymarin was procured from Yucca Enterprises (Mumbai, India). Orthophosphoric acid, ethanol, methanol, chloroform, acetonitrile, carbon tetrachloride, and Tween 20 were obtained from Hi-Media Laboratory Ltd. (Mumbai, India). Liver function test kits and the TBARS assay kit were purchased from Aspen Laboratories (Delhi, India). All chemicals/reagents used in this study were of analytical or HPLC grade.

### 3.2. Preparation of Silymarin Phytosomal Complex

Silymarin-phospholipid complexes (silymarin phytosomes) were prepared by the solvent evaporation technique, as previously described [34]. In brief, accurately weighed quantities of silymarin and soybean phosphatidylcholine (SPC) were placed in a 200 mL flask and dissolved in 50 mL ethanol. Silymarin-phospholipid complexes were prepared in different weight/weight ratios (1:1. 1:2 and 1:3). The solution was then refluxed at 65 °C, 70 °C, or 75 °C with the help of a rotary evaporator for 2 h. The resultant solution was concentrated in order to attain a thin lipid film. The obtained silymarin-phospholipid phytosomal complex was dried under vacuum to remove any traces of the solvent. The dried silymarin-phospholipid phytosomal complex was then transferred into light-resistant glass vials, purged with nitrogen gas and stored at room temperature.

### 3.3. Design of Experiments

A full factorial design (Design Expert^®^ software; Version 11.0.3.0) was utilized to investigate the impact of two independent formulation factors, namely, drug:phospholipid ratio (X_1_, w:w) and reaction temperature (X_2_, °C), on two dependent responses, namely, particle size (Y_1_) and drug content (Y_2_). The two independent variables (X_1_ and X_2_) were studied at three levels, denoted by the letters -1 (low), 0 (middle), and +1 (upper), resulting in a 3^2^-factorial design with nine independent experimental runs (Table 1). The results of the experiments were analyzed implementing a mathematical model defined by the polynomial equation given below:

Y= b_0_ + b_1_X_1_ + b_2_X_2_ + b_12_X_1_X_2_ + b_11_X_1_^2^ + b_22_X_2_^2^

where, Y is the dependent response and b is the regression coefficient of the independent variable X. X_1_ and X_2_ are the main factors, while X_1_X_2_ represents the interaction between main factors. X_1_^2^ and X_2_^2^ are the polynomial terms.

### 3.4. Evaluation of Silymarin Phytosomes

#### 3.4.1. Average Particle Size, Polydispersity Index and Zeta Potential

The average particle size, polydispersity index (PDI), and zeta potential of the prepared phytosomal formulations were determined by dynamic light scattering (DLS) and electrophoretic light scattering (ELS) techniques, respectively, using Malvern Zetasizer Nano-ZS (ZEN3600, Malvern Instrument Ltd., Malvern, UK) [35].

#### 3.4.2. Surface Morphology

The surface morphology of the optimized silymarin phytosomal formulation was studied by scanning electron microscopy (SEM) and transmission electron microscopy (TEM). The SEM sample was prepared onto double-sided adhesive tape with phytosomal powder spread over it, placed on an aluminum stub, and observed using JEOL-JSM 6380LA SEM (JEOL Ltd., Tokyo, Japan) [36]. For TEM, the phytosomal powder sample was diluted in distilled water (1:20) and sonicated using a probe sonicator (Vibra-Cell™ Sonicator, Newtown, CT, USA) for 3 min. The sonicated sample was cast on a 300 mesh copper grid (carbon type-B) and allowed to adsorb as a thin liquid film by removing excess sample using filter paper, stained with uranyl acetate solution (2% *w*/*v*), and dried overnight under vacuum. This stained liquid film was observed under JEOL-JEM-100S TEM (JEOL Ltd., Tokyo, Japan) with an operating voltage 200 kV [37].

#### 3.4.3. Estimation of Drug Content

The amount of silymarin incorporated within the formulated phytosomal complex was estimated by a spectrophotometric method. Briefly, an accurately weighed quantity of phytosomal complex (5 mg) was dispersed in 5 mL of chloroform; where the formulated phytosomes dissolve in chloroform, while non-complexed silymarin remains insoluble. Upon filtering the dispersion, non-complexed silymarin was separated as a solid residue, dried and re-dissolved in methanol [38]. The concentration of free non-complexed silymarin was determined spectrophotometrically at λ_max_ of 286 nm using UV-visible spectrophotometer (Shimazu, Tokyo, Japan). Drug content (%) was calculated using the following formula:

$$\mathrm{Drug}\;\mathrm{content}\;\left(\%\right)=\frac{\mathrm{Total}\;\mathrm{amount}\;\mathrm{of}\;\mathrm{Silymarin}-\mathrm{amount}\;\mathrm{of}\;\mathrm{free}\;\mathrm{Silymarin}}{\mathrm{Total}\;\mathrm{amount}\;\mathrm{of}\;\mathrm{Silymarin}}\;\times \;100$$

#### 3.4.4. Fourier Transform Infrared Spectroscopy (FTIR)

The chemical interaction between phytosomal components was studied using an infrared (IR) spectra matching approach using a FTIR spectrometer (Alpha Bruker, Berlin, Germany). The IR spectrum of the pure silymarin, soybean phosphatidyl choline (SPC), physical mixture of silymarin and SPC (1:1), and optimized silymarin phytosomal formulation was obtained by scanning within the wavelength range of 4000 to 500 cm^−1^ [39].

#### 3.4.5. Differential Scanning Calorimetry (DSC)

Thermograms of silymarin, SPC, physical mixture of silymarin and SPC, and optimized silymarin phytosomal formulation were recorded on differential scanning colorimeter (TGA/DSC-SDT Q600, TA Instruments, New Castle, DE, USA). The thermal behavior was investigated by heating 2 mg of the individual samples at a heating rate of 10 °C/min from 25 °C to 400 °C in a covered sample pan under a nitrogen purge of 60 mL/min [40].

#### 3.4.6. X-ray Powder Diffractometry (XRD)

X-ray diffractograms of silymarin, SPC, and optimized silymarin phytosomal formulation were recorded in X-ray diffractometer (Rigaku mini flex 600, Hokkaido, Japan) to study the molecular crystallinity. The instrument was adjusted at 40 kV tube voltage, 40 mA tube current, and 5–50° scanning angle of 2θ with a 1°/min step width [41].

### 3.5. Solubility Study

The apparent solubility of the samples was determined by adding excess amounts of silymarin, and optimized silymarin phytosomal formulation to 5 mL of distilled water or n-octanol into sealed glass containers at 25 ± 1 °C. The solution was then agitated for 24 h and further centrifuged for 30 min at 5000 rpm. Further, 1 mL of the filtrate was diluted up to 10 mL with respective solvents and then analyzed spectrophotometrically at λ_max_ of 286 nm [27].

### 3.6. In Vitro Dissolution Study

The in vitro dissolution profile of the silymarin phytosomal complex was carried out using the dialysis bag method to determine the drug release from the formulation. Briefly, 10 mg of pure silymarin and a definite weight of optimized silymarin phytosomal formulation, equivalent to 10 mg silymarin, were placed in the dialysis bags. The bags were placed in glass vials enclosing 100 mL of phosphate buffer pH 7.4. The glass vials were agitated at 50 rpm and 37 ± 1 °C. At predetermined time points, 2 mL samples were withdrawn from the glass vials and were interchanged with an equal volume of fresh buffer to retain the sink condition. The collected samples were filtered, suitably diluted, and analyzed using a UV spectrophotometer (Shimadzu, Tokyo, Japan) at 286 nm. To study the drug release mechanism from the formulation, the in vitro release data was fitted into different in vitro kinetic release models.

### 3.7. In Vivo Studies

#### 3.7.1. Animals

Male Wistar rats (175–200 g) were used in this investigation. All animal experiments were reviewed and approved by the Animal Ethics Committee of N.G.S.M Institute of Pharmaceutical Sciences (NGSMIPS/IAEC/140). The rats were adapted to laboratory conditions by housing in groups of 7–8 in rat-breeding plastic tubs with stainless steel straight wired lid, at 22 ± 2 °C and 50 ± 10% relative humidity with a 12/12 h light-dark cycle for 10 days prior to experiments.

#### 3.7.2. In Vivo Hepatoprotective and Antioxidant Activity Studies

Wistar rats were randomly categorized into 4 groups (n = 6). The first group (negative control group) was treated orally with an aqueous solution of Tween 20 (1% *v*/*v*) for 7 days. The second group received an aqueous solution of Tween 20 (1% *v*/*v*) orally for 7 days followed by a single i.p. dose of a mixture of carbon tetrachloride (CCl_4_) and olive oil (1:1.5 mL/kg) on the 7th day, and served as a positive control group. The third group was treated orally with silymarin suspension (100 mg/kg/day) for 7 days, followed by a single i.p. dose of a mixture of CCl_4_ and olive oil on the 7th day. The last group was treated orally with optimized silymarin phytosomal suspension (100 mg silymarin/kg/day) for 7 days, followed by a single i.p. dose of a mixture of CCl_4_ and olive oil on the 7th day. At 24 h post CCL_4_ intoxication, blood samples were collected, centrifuged and sera samples were separated. Liver function test (LFT) was assessed by quantitative determination of liver marker enzymes such as serum glutamate pyruvate transaminase (SGPT), serum glutamate oxaloacetate transaminase (SGOT), serum alkaline phosphatase (SALP), and total bilirubin.

For biochemical estimation of liver antioxidant enzymes, animals were euthanized post blood samples collection. The livers were dissected, rinsed with ice-cold saline, and were subjected to homogenization with 0.1M Tris HCl buffer (pH 7.4). The liver homogenate was centrifuged, and the supernatant was subjected to glutathione reductase (GRD), reduced glutathione (GSH), glutathione peroxidase (GPx), glutathione S transferase (GST), superoxide dismutase (SOD), and catalase (CAT). Lipid peroxidation was also estimated by quantifying the amount of malondialdehyde (MDA) in the liver homogenates using thiobarbituric acid reactive substance (TBARS) assay [30]. This assay involves the reaction of MDA with thiobarbituric acid (TBA) forming a pink chromogen (TBARS), which is measured at 530 nm. The concentration of MDA is expressed as nM of MDA/mg of protein.

#### 3.7.3. Histopathological Studies

For histopathological observation, the dissected animal livers were stored in 10% *v*/*v* neutral buffered formalin. Later, the haematoxylin and eosin-stained liver sections were prepared and observed under Zeiss Primo Star microscope (Carl-Zeiss, Oberkochen, Germany).

#### 3.7.4. In Vivo Pharmacokinetic Studies

For pharmacokinetic studies, Wistar rats were categorized into two groups (*n* = 6); control group receiving pure silymarin (100 mg/kg) and treatment group receiving optimized silymarin phytosomal formulation (100 mg silymarin/kg). At scheduled time points post-administration (0.5, 1, 2, 3, 4, 5, 6, 8, and 12 h), blood samples (500 μL) were collected from the retro-orbital plexus in heparinized tubes and centrifuged at 3000 rpm for 10 min to obtain plasma. Then, 200 μL of separated plasma samples was mixed with 1 mL methanol. The mixture was heated at 75 °C for 30 min and was further centrifuged at 4000 rpm for 30 min using a cooling centrifuge. The supernatant was filtered using a 0.22 µm membrane filter and drug concentration was quantified by HPLC analysis, as described previously [42]. Pharmacokinetic parameters of silymarin, including maximum plasma concentration (C_max_), time required to reach a maximum concentration (t_max_), elimination half-life (t_1/2_), elimination rate constant (K_el_), volume of distribution (V_d_), clearance (Cl), and area under the plasma concentration-time curve (AUC_0–24h_) were calculated using PK Solver 2.0 software. Furthermore, the bioavailability of optimized silymarin phytosomal formulation relative to that of pure silymarin was calculated.

### 3.8. Statistical Analysis

All data are presented as mean ± SD. Statistical analysis was performed using Student’s *t*-test and one way analysis of variance (ANOVA). Statistical significance was defined at *p* values less than 0.05.

## 4. Conclusions

The present study manifested the potential of amphiphilic phospholipid-based phytosomes for enhancing the solubility, absorption, oral bioavailability, and in vivo hepatoprotective activity of the polyphenolic phyto-constituent, silymarin. Silymarin phytosomes were prepared using the solvent evaporation method and optimized using a full factorial design. The optimized silymarin phytosomal formulation efficiently enhanced the aqueous solubility of silymarin and sustained in vitro drug release for up to 24 h compared to the plain drug. In addition, in a CCl_4_-induced hepatotoxicity rat model, compared to the plain drug, the optimized silymarin phytosomal formulation showed superior hepatoprotective effects as manifested by efficient restoration of normal levels of antioxidant enzymes and ameliorating all cellular changes induced by CCl_4_-intoxication. Most importantly, the optimized silymarin phytosomal formulation significantly improved silymarin oral bioavailability as evidenced by a ~6-fold increase in systemic bioavailability compared to pure silymarin. Collectively, our results emphasize the utility of phospholipid-based phytosomes in improving the aqueous solubility, oral bioavailability, and thereby the pharmacological activities of poorly soluble phyto-constituents.

## Figures and Tables

**Table 1:** Experimental design matrix of the full factorial design with experimental results.

X_1_ (*w*:*w*)	X_2_ (°C)	Y_1_ (nm)	Y_2_ (*w*/*w*%)
+1	−1	494.2 ± 6.64	67.3 ± 2.64
−1	+1	329.6 ± 4.21	81.7 ± 3.47
0	−1	402.8 ± 3.87	75.6 ± 2.92
+1	+1	395.8 ± 2.45	78.7 ± 2.43
0	+1	313.1 ± 4.69	83.5 ± 3.38
−1	−1	436.1 ± 5.92	70.3 ± 2.79
0	0	220.2 ± 1.27	92.4 ± 3.51
−1	0	255.3 ± 1.52	89.2 ± 3.68
+1	0	307.8 ± 3.95	85.4 ± 3.74

X_1_: drug:phospholipid ratio; X_2_: the reaction temperature; Y_1_: particle size; and Y_2_: drug content. Data represents mean ± SD of three independent experiments.

**Table 2:** Solubility of pure silymarin and optimized silymarin phytosomal formulation in water and phosphate buffer pH 7.4.

Medium	Solubility (µg/mL)	
	Pure Silymarin	Optimized Silymarin Phytosome
Water	45.73 ± 2.4	358.79 ± 9.4
n-octanol	129.29 ± 1.5	568.54 ± 8.5

Data represent mean ± SD of three independent experiments.

**Table 3:** Hepatic marker enzymes levels following treatment with plain silymarin and optimized silymarin phytosomal formulation in CCl_4_-intoxicated rat model.

Hepatic Antioxidant Enzyme	Group—I (Normal Control)	Group—II (CCl_4_-Intoxicated Rats)	Group—III (Plain Silymarin)	Group—IV (Optimized Silymarin Phytosomes)
SGPT (U/L)	42.77 ± 1.82 **	134.37 ± 3.61	95.68 ± 3.56 **	57.35 ± 2.73 **
SGOT (U/L)	38.22 ± 2.71 **	97.76 ± 3.38	75.19 ± 3.22 *	46.88 ± 2.25 **
SALP (U/L)	141.53 ± 2.26 **	267.64 ± 3.29	221.77 ± 3.41 *	159.43 ± 3.55 **
Total bilirubin (mg/dL)	0.66 ± 0.03 **	1.41 ± 0.02	0.95 ± 0.02 **	0.72 ± 0.01 **

Data are mean ± SD (*n* = 6). * *p* < 0.05 and ** *p* < 0.01 vs. CCl_4_-intoxicated rats. SGPT: serum glutamate pyruvate transaminase; SGOT: serum glutamate oxaloacetate transaminase; SALP: serum alkaline phosphatase.

**Table 4:** Effect of plain silymarin and optimized silymarin phytosomes on antioxidant enzymes.

Hepatic Antioxidant Enzyme	Group—I (Normal Control)	Group—II (CCl_4_-Intoxicated Rats)	Group—III (Plain Silymarin)	Group—IV (Optimized Silymarin Phytosomes)
GSH (nmol/mg protein)	49.16 ± 3.99 **	18.86 ± 1.28	29.37 ± 2.34 *	41.22 ± 2.15 **
GPx (nmol/mg protein)	332.23 ± 4.91 **	193.76 ± 3.71	244.35 ± 4.27 *	302.43 ± 3.33 **
GST (nmol/mg protein)	296.43 ± 4.73 **	165.28 ± 3.45	210.55 ± 4.28	264.88 ± 4.23 **
GRD (nmol/mg protein)	21.16 ± 1.54 **	7.89 ± 0.95	13.26 ± 1.54	18.47 ± 1.21 **
SOD (U/mg protein)	7.41 ± 0.11 **	4.21 ± 0.14	5.03 ± 0.22	6.21 ± 0.03 **
CAT (U/mg protein)	212.85 ± 2.87 **	95.46 ± 3.42	140.15 ± 3.76 *	187.29 ± 2.58 **

Data represent mean ± SD (*n* = 6). * *p* < 0.05 and ** *p* < 0.01 vs. CCl_4_-intoxicated rats. GSH: glutathione; GPx: glutathione peroxidase; GST: glutathione S transferase; GRD: glutathione reductase; SOD: superoxide dismutase; and CAT: catalase.

**Table 5:** Pharmacokinetic parameters of pure silymarin and (100 mg/kg, p.o.) and optimized silymarin phytosomes (~100 mg/kg silymarin, p.o).

Pharmacokinetic Parameters	Pure Silymarin	Optimized Silymarin Phytosomes
C_max_ (µg mL^−1^)	0.40 ± 0.10	1.10 ± 0.21 **
T_max_ (h)	4.0	6.0 **
AUC_0-t_ (µg mL^−1^ h)	2.80 ± 0.71	45.76 ± 1.41 **
AUC_0-∞_ (mL^−1^ h)	3.84 ± 0.91	22.33 ± 2.13
Elimination half-life (t_1/2_) (h)	6.01 ± 0.70	12.31 ± 0.96 **
Elimination rate constant (K_el_) (h^−1^)	0.12 ± 0.02	0.06 ± 0.01 **
Mean residance time (MRT) (h)	9.44 ± 1.10	20.43 ± 1.76 **
Clearance (Cl) (mL·h^−1^)	26.03 ± 1.93	4.48 ± 0.77 **
Volume of distribution (V_d_) (mL^−1^)	255.48 ± 12.33	79.53 ± 8.11 **

Data are mean ± SD (n = 6). ** *p* < 0.01.

## Data Availability

Data is contained within the article and supplementary material.

## Supplementary Materials

The following supporting information can be downloaded at: https://www.mdpi.com/article/10.3390/ph15070790/s1, Figure S1: Particle size distribution of optimized silymarin phytosomal formulation; Figure S2: Zeta potential of optimized silymarin phytosomal formulation.
