# Supplementary material for: Phytosomes as a Plausible Nano-Delivery System for Enhanced Oral Bioavailability and Improved Hepatoprotective Activity of Silymarin
Source: Pharmaceuticals (Basel). 2022 Jun 24;15(7):790. doi: 10.3390/ph15070790 (PMC9318442; doi:10.3390/ph15070790)
Supplement: Supplementary file 1 [file pharmaceuticals-15-00790-s001.zip › pharmaceuticals-1784153-supplementary.pdf]

Phytosomes as a Plausible Nano-Delivery System for Enhanced Oral Bioavailability and Improved Hepatoprotective Activity of Silymarin

Ravi Gundadka Shriram [1], Afrasim Moin [2], Hadil Faris Alotaibi [3], El-Sayed Khafagy [4,5], Ahmed Al Saqr [4], Amr Selim Abu Lila [2,6,*] and Rompicherla Narayana Charyulu [1,*]

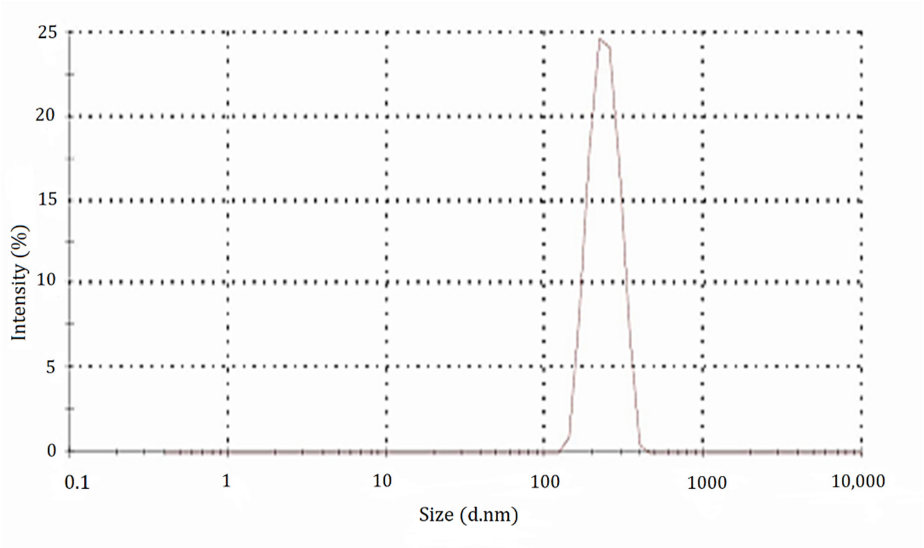

Figure S1. Particle size distribution of optimized silymarin phytosomal formulation

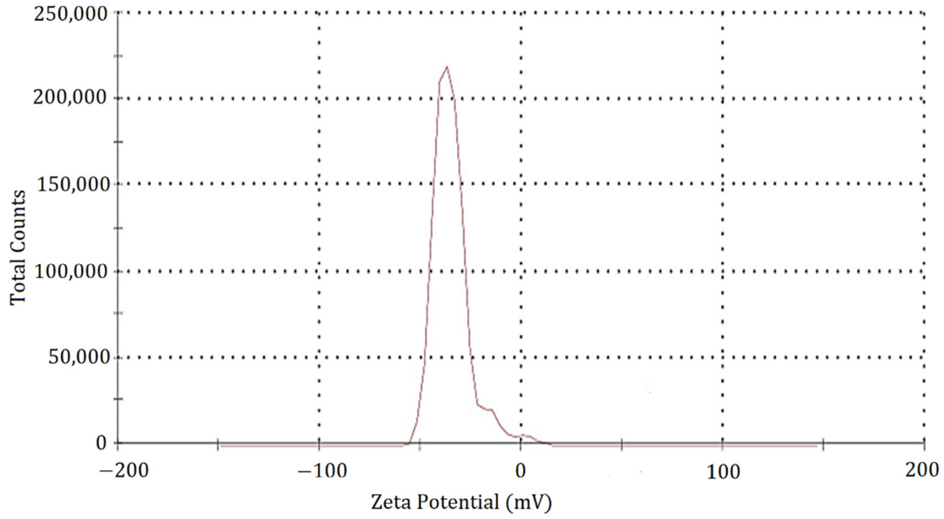

Figure S2. Zeta potential of optimized silymarin phytosomal formulation.
